# Infraocclusion in the Primary and Permanent Dentition—A Narrative Review

**DOI:** 10.3390/medicina60030423

**Published:** 2024-03-01

**Authors:** Paulina Adamska, Hanna Sobczak-Zagalska, Marcin Stasiak, Łukasz Jan Adamski, Dorota Pylińska-Dąbrowska, Sandra Barkowska, Adam Zedler, Michał Studniarek

**Affiliations:** 1 Division of Oral Surgery, Faculty of Medicine, Medical University of Gdańsk, 7 Dębinki Street, 80-211 Gdańsk, Poland; 2 Department of Pediatric Dentistry, Faculty of Medicine, Medical University of Gdańsk, 18 Orzeszkowa Street, 80-204 Gdańsk, Poland; hanna.sobczak-zagalska@gumed.edu.pl; 3 Division of Orthodontics, Faculty of Medicine, Medical University of Gdańsk, Al. Zwycięstwa 42c, 80-210 Gdańsk, Poland; marcin.stasiak@gumed.edu.pl; 4 Individual Dental Practice Łukasz Adamski, 3b Stawiska, 83-431 Stary Bukowiec, Poland; lukaszadamski@gumed.edu.pl; 5 Department of Dental Prosthetic, Faculty of Medicine, Medical University of Gdańsk, 18 Orzeszkowa Street, 80-204 Gdańsk, Poland; dorota.pylinska-dabrowska@gumed.edu.pl; 6 Individual Dental Practice Sandra Barkowska, 25 Rubinowa Street, 83-422 Lubań, Poland; 7 Department of Radiology, Faculty of Medicine, Medical University of Gdańsk, 17 Smoluchowskiego Street, 80-216 Gdańsk, Poland

**Keywords:** deciduous tooth, hypodontia, infraocclusion, infraposition, reinclusion, tooth sinking, tooth submerging

## Abstract

The gradual movement of a tooth away from the occlusal plane is called infraocclusion or reinclusion. Reincluded teeth are most often deciduous molars, and permanent teeth are less frequently affected. Depending on the level of the infraocclusion, the severity of the disorder is classified as mild, moderate, or severe. The etiology of the phenomenon is not fully known. Tooth submerging can lead to serious complications, such as abnormal position of adjacent teeth, displacement of the bud of the permanent successor, shortening of the dental arch, or developmental disturbances of alveolar process. Early diagnosis of the tooth infraocclusion and regular monitoring of its progression help to avoid serious permanent sequelae. The treatment of reinclusion often involves only observation. However, in some cases, the therapeutic procedure requires interdisciplinary treatment by specialists from various fields of dentistry. This study presents current methods of diagnosis and treatment of patients with submerged teeth.

## 1. Introduction

The phenomenon of teeth submerging, clinically known as reinclusion (the gradual movement of a tooth from the occlusal plane), represents a significant challenge within dental medicine, affecting both primary and permanent dentitions. Historically, the condition has not only perplexed clinicians but also stimulated extensive research into its etiology, diagnosis, and management strategies. Other terms for this phenomenon used in literature are half retention, infraposition, infraocclusion, secondary retention, reimpaction, and submerged, reincluded, depressed, or embedded teeth [1,2].

Tooth ankylosis, characterized by the fusion of tooth cementum and dentine with alveolar bone, plays a pivotal role in the submerging of teeth. This pathological union impedes the natural vertical movement of the tooth in response to the dynamic changes in the jaw, leading to its gradual submersion relative to the occlusal plane. Recent studies suggest that ankylosis may result from a combination of factors, including trauma, genetic predisposition, and local environmental conditions, contributing to the replacement resorption process. Histological examination of the root surfaces of extracted reincluded teeth showed the presence of sites of ankylosis on most of these roots. The area of ankylosis was often limited to such an extent that it did not cause radiological and clinical symptoms in the form of a metallic percussion tone [3]. Bone inertia, another crucial factor, refers to the loss of bone’s adaptive growth capacity in the vicinity of an affected tooth. This condition, hypothesized by Brabant, implies a localized failure in bone remodeling, essential for accommodating the shifting positions of teeth during normal development. The stagnation of alveolar bone growth around a reincluded tooth not only contributes to its submergence but also impacts the overall dental arch development. Beyond ankylosis and bone inertia, a spectrum of hereditary, hormonal, and environmental factors has been implicated in the etiology of tooth submerging. Genetic studies have pointed to the heritability of certain dental conditions that predispose individuals to ankylosis and infraocclusion, suggesting a complex interplay between genes and environmental triggers. Hormonal imbalances, particularly those affecting growth and developmental pathways, may also influence the risk of tooth submerging. For instance, conditions that alter the normal regulation of bone metabolism can indirectly affect the process of tooth eruption and positioning [1,2].

Environmental factors, including the impact of chronic dental infections, exposure to certain chemicals, and habitual parafunctions, have been observed to alter the local oral environment in ways that may predispose teeth to submerging or exacerbate the submerging of teeth. Additionally, nutritional deficiencies and systemic diseases that affect bone and dental health can contribute to the development of this condition [4,5,6,7,8,9,10].

Despite advancements in dental science, the underlying mechanisms and optimal treatment protocols for infraocclusion remain subjects of ongoing investigation. This article endeavors to illuminate the complex and multidimensional aspects of tooth reinclusion, delving into the array of factors influencing its occurrence, progression, and management. In the pursuit of advancing patient care, it is imperative to recognize and integrate the latest trends and technological innovations that have emerged in the dental field. These advancements not only offer a deeper understanding of the underlying mechanisms of infraocclusion but also pave the way for developing more effective diagnostic tools and treatment modalities.

## 2. Diagnostics and Treatment

Infraocclusion most often occurs in primary teeth, but it can also affect permanent teeth. The pathology is mainly associated with the first and second mandibular primary molars and may be accompanied by the absence of a permanent successor. In the permanent dentition, the infraposition is also more often reported in the first and second molars of the mandible. Submerging of teeth in the lower jaw can cause dilaceration, laceration, and bending of the root. Such root abnormalities are only observed at the stage of root development when a submerging tooth encounters a barrier of hard bone tissue. A similar situation occurs when the tooth roots rest against the cortical layer of the floor of the maxillary sinus [4,5,11,12].

**Figure 1:**
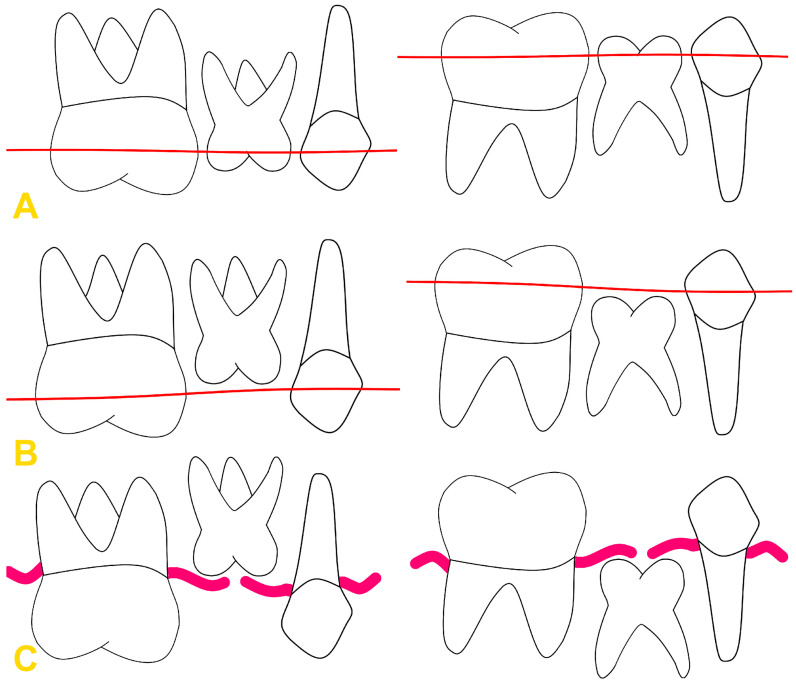
Pytlik classification: (**A**) phase A1, (**B**) phase A2, and (**C**) phase B. Thin red line—half the height of the crowns of adjacent teeth. Thick red line—gingival margin.

The incidence of reinclusion varies among populations and is reported to range from 1% to 39% [4,5,11,12,13]. Completely or partially erupted teeth may undergo reinclusion. The element that distinguishes a reincluded tooth from an impacted tooth is the presence of a canal lined with epithelium, which communicates the tooth with the oral cavity. The classification proposed by Pytlik is based on the degree of tooth infraposition, dividing the reinclusion into partial and complete (Figure 1) [14]. Partial reinclusion includes phase A1, in which the tooth crown reaches half the height of the crowns of adjacent teeth, and phase A2, in which the crown of the embedded tooth is located between half the height of the crowns of adjacent teeth and the cervix. Complete tooth immersion—phase B—is described when the tooth crown is below the gingiva. Infraocclusion can also be divided into mild (immersion of 1–2 mm), moderate (infraposition of 2–3 mm), and severe (immersion of more than 3 mm) [4,6,14,15].

Diagnosis of reinclusion includes medical history and physical and radiological examination. The information obtained from medical interview helps to determine the progression of the process as well as its possible cause (e.g., tooth trauma). Additionally, it allows us to differentiate the primary failure of eruption (PFE) in which the tooth does not appear in the oral cavity due to a disturbed eruption mechanism, from a secondary disorder, in which the eruption of a tooth, which is already visible in the dental arch, is arrested. PFE is less common than tooth reinclusion. Its prevalence is about 0.06% [16]. PFE refers to teeth with partial or complete lack of eruption due to the disturbed mechanics of this process without specific identifiable local or systemic involvement. It primarily affects posterior teeth and all teeth distal to the most anteriorly affected tooth. As a result, posterior unilateral/bilateral open bite occurs. It is important not to use orthodontic extrusion on teeth with primary failure of eruption, as it will cause ankylosis of these teeth [17].

Failure of an eruption is often a major diagnostic problem. It may coexist with systemic or syndromic disorders, such as cleidocranial dysplasia, odontodysplasia, and GAPO syndrome (growth delay–alopecia–pseudoanodontia–optic atrophy syndrome). However, it more often occurs in the form of reinclusion, PFE, or mechanical failure of eruption (MFE). Careful examination must be undertaken to make a correct diagnosis as the treatment procedures vary between these disorders [17].

The infraocclusion of the third permanent molar, often referred to an impacted wisdom tooth, is primarily attributed to spatial constraints within the dental arch, directly behind the second permanent molar. This condition is further exacerbated by the aberrant positioning and angulation of the wisdom teeth. Factors contributing to these spatial limitations include the evolutionary trend towards smaller jaw sizes in modern humans, which do not accommodate the proper eruption and alignment of third molars [18].

A metallic, ankylotic percussion sound may be heard during vertical percussion examination. The presence of carious lesions, fillings, and tooth wear facets on reincluded teeth indicates that these teeth have previously been erupted [3]. Adjacent teeth may be inclined towards the reincluded tooth. Moving the teeth towards the reincluded tooth disrupts the occlusal plane. This causes the Godon effect in the opposing dental arch and involves the slow eruption of opposing teeth. Moreover, it might make it difficult to clean the teeth and thus cause caries or periodontitis. The tilting of the teeth adjacent to the gap causes the formation of dental pockets. The communication through the epithelial canal with the oral environment may cause inflammation, including complications manifesting as submucosal abscesses. This is especially dangerous in patients of developmental age. Children have less bone calcification, wide marrow cavities, rich vascularization in the bone marrow, and the presence of tooth buds. The maxillary sinus is also partially developed and there is a large amount of spongy bone. This causes inflammation to spread to adjacent anatomical spaces. The most dangerous complications are orbital complications (orbital abscess and empyema), cerebral complications, and progression of inflammation to the mediastinum [19].

The affected primary tooth may be accompanied by the absence of a permanent tooth bud or, if present, the successor may be impacted due to obturation. In the case of severe reinclusion, the teeth may intrude towards the maxillary sinus or cause pressure on the lower alveolar nerve (Vincent’s sign—sensory disturbances and numbness of the lower lip on the side of the disorder). A tooth displaced into the maxillary sinus may cause inflammation of the maxillary sinus or imply the development of cystic tumors. Infraoclussion of teeth may cause impairment of the stomatognathic system and the occurrence of malocclusions at the dental and dentoalveolar level—open bite, crossbite, overhanging bite, and others [4,5,20,21,22,23].

**Figure 2:**
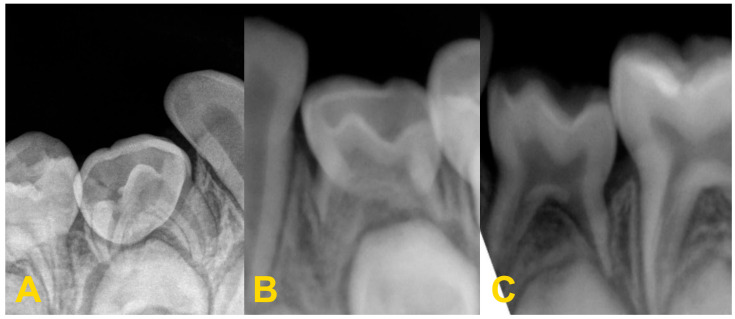
Radiological imaging—dental X-ray of teeth nos.: (**A**) 84, (**B**) 74, and (**C**) 74.

**Figure 3:**
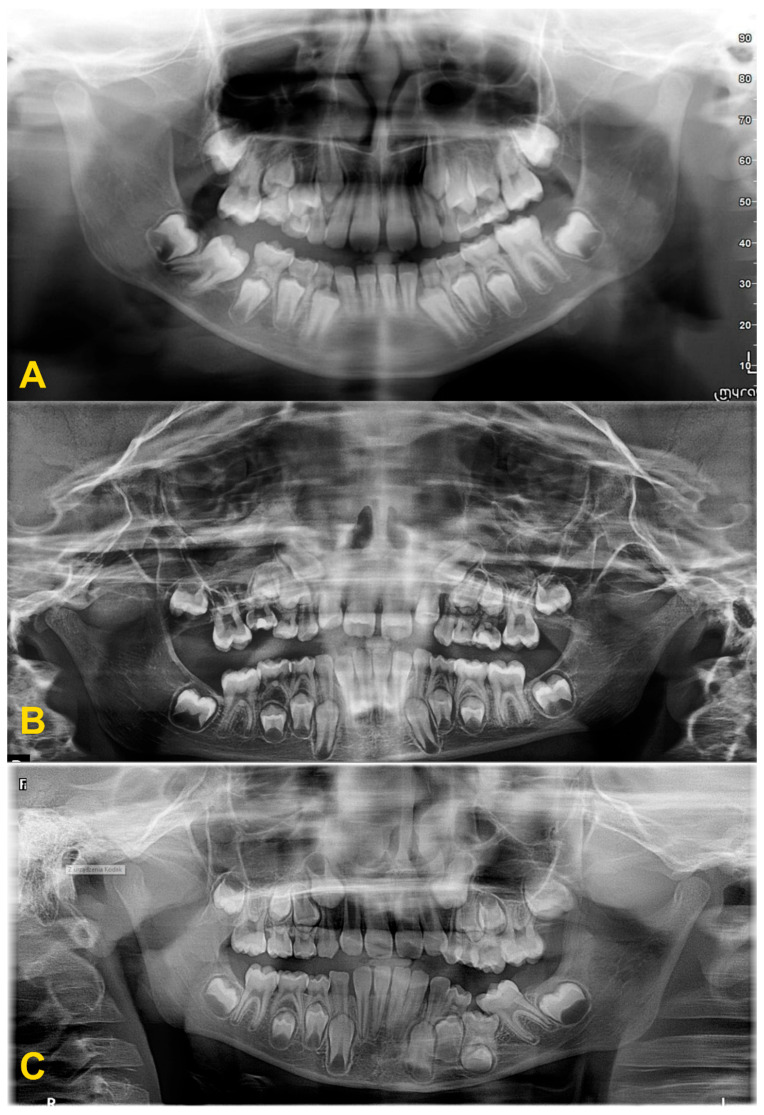
Panoramic X-ray examinations: (**A**) mild infraocclusion of tooth no. 55 (immersion of 1–2 mm); (**B**) moderate infraocclusion of tooth no. 55 (infraposition of 2–3 mm), displacement and growth delay of permanent successor, and angulation of the adjacent teeth with loss of space; and (**C**) severe infraocclusion of tooth no. 75 (immersion of more than 3 mm), displacement and growth delay of permanent successor, severe angulation of the adjacent teeth with loss of space (the same patient as in Figure 6C).

**Figure 4:**
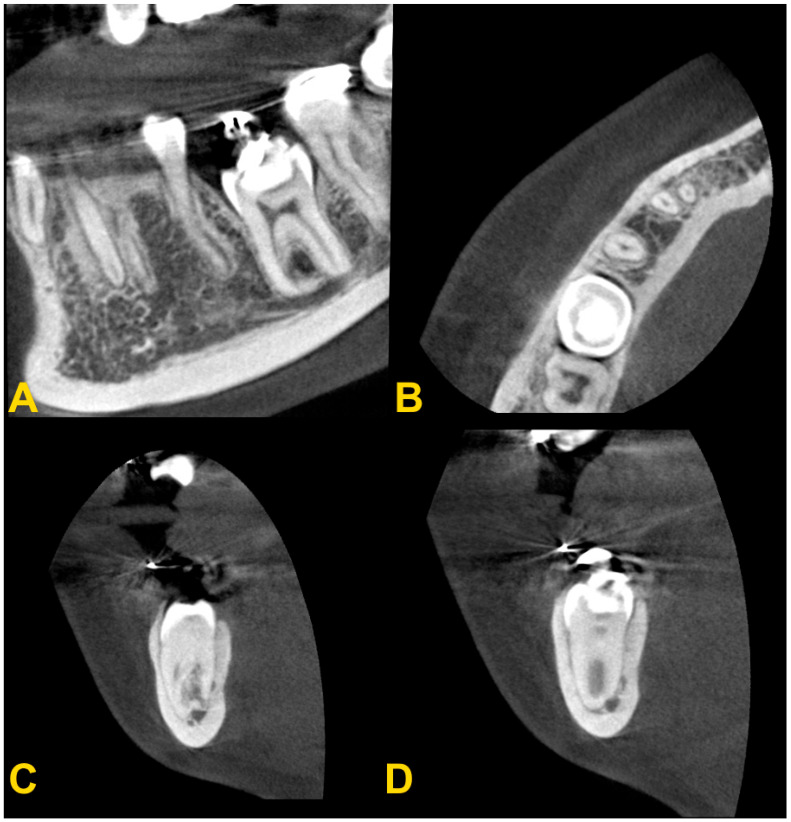
Cone-beam computed tomography examination of tooth no. 36 with infraocclusion: (**A**) panoramic reconstruction, (**B**) axial view, (**C**) transectal view through the mesial root, and (**D**) transectal view through the distal root (the same patient as in Figure 6D).

**Figure 5:**
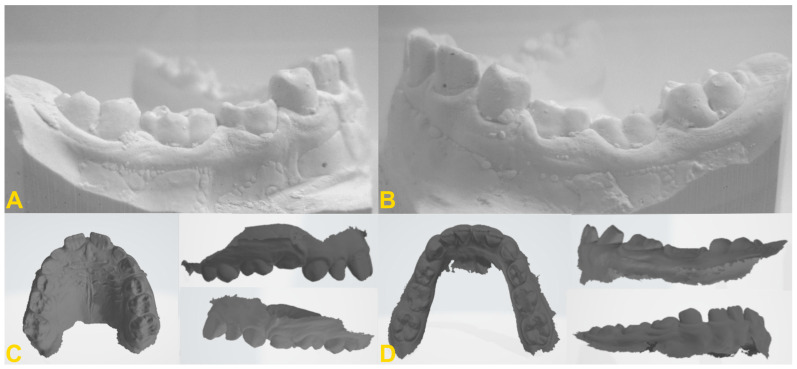
Diagnostic models of the mandible (**A**,**B**) and intraoral scans (**C**,**D**): (**A**) right side—infraposition of primary molars in the mandible; (**B**) left side—infraposition of primary molars in the mandible with angulation of the adjacent teeth; (**C**) maxilla—occlusal view and right- and left-side view—infraposition of teeth nos. 54, 55, and 65; and (**D**) mandible—occlusal view and left- and right-side view—infraposition of teeth nos. 74, 75, 84, and 85 (the same patient as in Figure 9).

**Figure 6:**
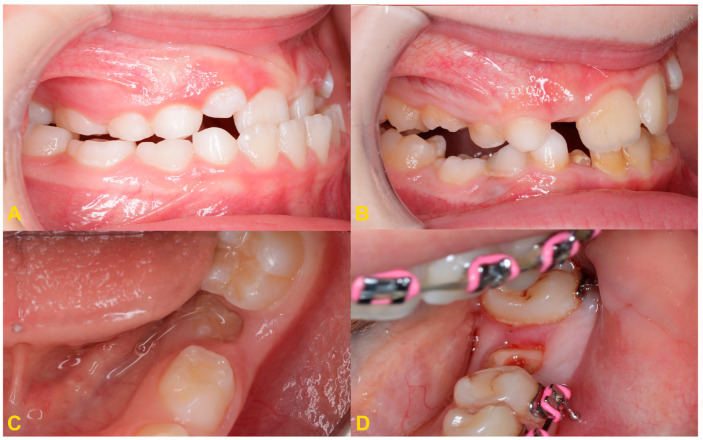
Intraoral photography: (**A**) mild reinclusion of tooth no. 85 in the presence of a permanent successor—the patient requires observation; (**B**) moderate reinclusion of teeth nos. 55 and 85 in the absence of permanent successors, and presence of lateral open bite—the teeth require extraction; (**C**) severe reinclusion of tooth no. 75 in the presence of permanent successor (the same patient as in Figure 3C)—the tooth requires extraction; and (**D**) infraocclusion within the permanent dentition—tooth no. 36—the tooth should be removed (the same patient as in Figure 4).

**Figure 7:**
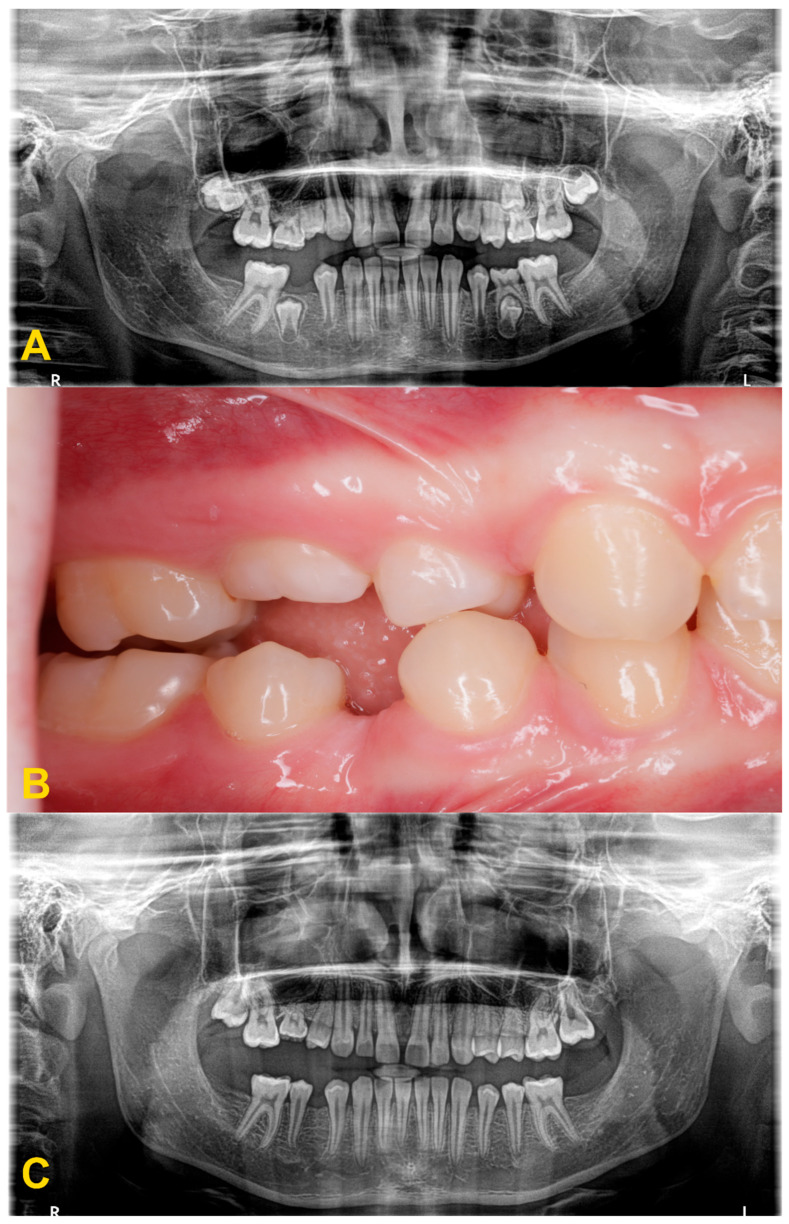
Progression of infraocclusion in tooth no. 55 in the absence of a permanent successor. Extraction is recommended due to the possible progression of the bone defect and inclination of adjacent teeth. Spontaneous exfoliation of tooth no. 75 with infraocclusion and self-correction of second premolar’s distoinclination. Missing teeth nos. 37 and 47. Failure of eruption of teeth nos. 17 and 27, surgical exposure is indicated. (**A**) panoramic X-ray, (**B**) intraoral photography, and (**C**) panoramic X-ray with 4-year follow-up.

**Figure 8:**
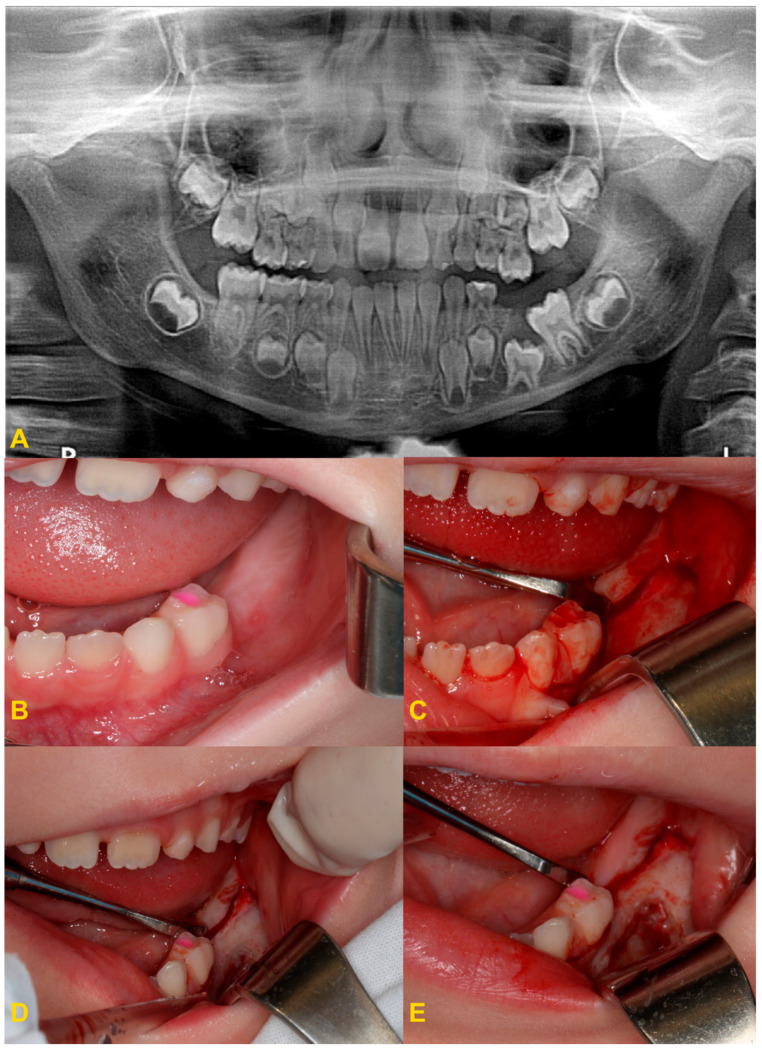

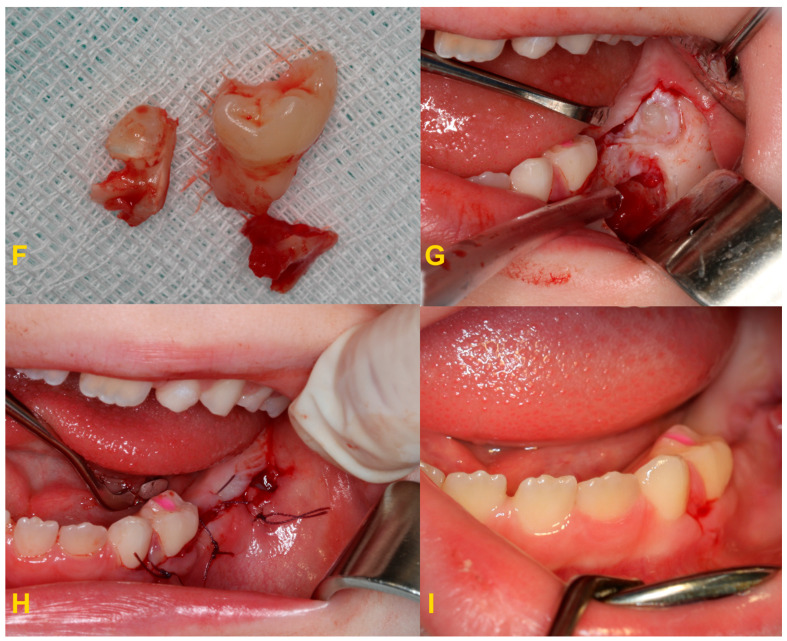
Severe reinclusion of tooth no. 75 causing infraposition of the tooth no. 36: (**A**) panoramic X-ray, (**B**) preoperative intraoral photography—teeth nos. 75 and 36 are missing in the oral cavity, (**C**) intraoral photography—after elevation of the mucoperiosteal flap, (**D**) intraoral photography—osteotomy, (**E**) intraoral photography—crown–root separation, (**F**) fragments of extracted tooth no. 75, (**G**) intraoral photography—osteotomy and exposure of tooth no. 36, (**H**) intraoral photography—stitching the wound, and (**I**) intraoral photography—healing on the 7th day after the procedure.

**Figure 9:**
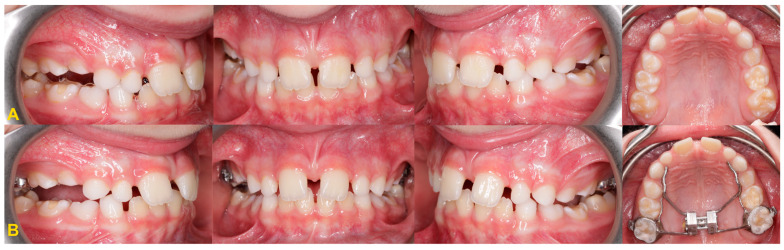
Moderate reinclusion of the teeth nos. 55 and 65, and severe reinclusion of tooth no. 54—use of the teeth as an anchorage in orthodontic treatment: (**A**) before treatment and (**B**) maxillary expansion with the Hyrax appliance (the same patient as in Figure 5C,D).

**Figure 10:**
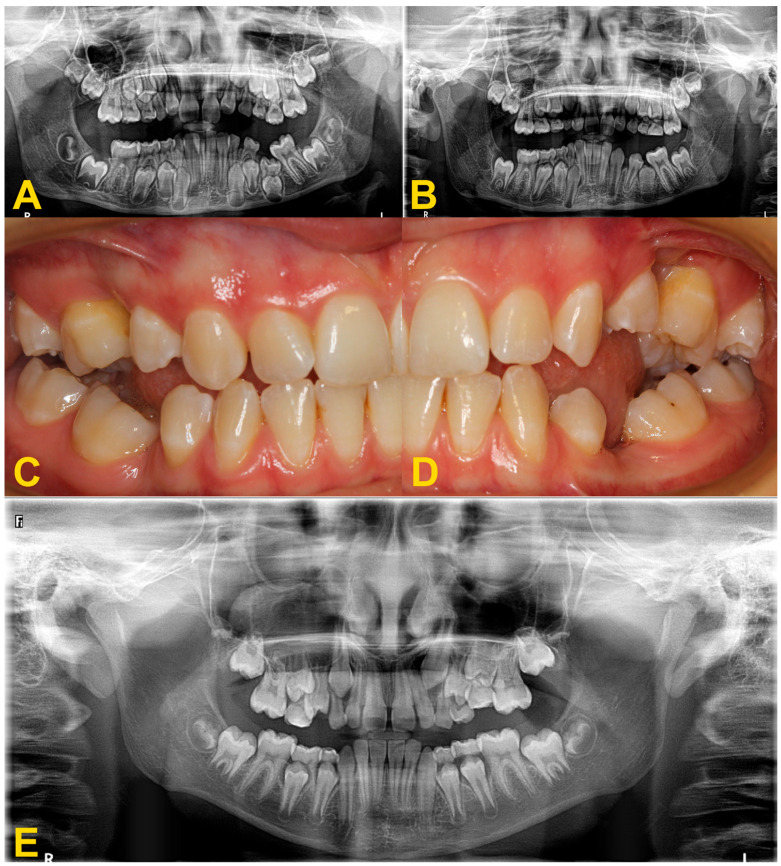
Disorders of the occlusal plane and displacement of the adjacent teeth: (**A**,**B**) panoramic X-rays—displacement of the adjacent teeth, bone defect and growth delay of the permanent successor due to the severe infraocclusion of tooth no. 75 before (**A**) and after the extraction of tooth no. 75 (**B**). Surgery was performed under general anesthesia with simultaneous extraction of teeth nos. 38 and 48 due to crowding. Lack of space for tooth no. 35, improvement of root development and position of tooth no. 35; (**C**,**D**) intraoral photography (another patient)—displacement of the adjacent teeth, disturbance of the occlusal plane with lateral open bite, and bone defect—situation after too-late removal of reincluded teeth with lack of permanent successors, (**E**) panoramic X-ray—moderate infraocclusion of tooth no. 65, displacement of the tooth no. 25 in relation to its antimere, incorrect position of the tooth no. 23 with suspected cyst, moderate infraocclusion of teeth nos. 74, 84, and 85, no resorption of the mesial root of tooth no. 85, mild infraocclusion of tooth no. 75.

The diagnosis of tooth infraocclusion is multifaceted, involving a combination of detailed medical history, clinical examination, and advanced imaging techniques. To extend the diagnosis and monitor the reinclusion process, radiological examinations (dental X-rays—Figure 2, panoramic X-rays—Figure 3, and cone-beam computed tomography (CBCT)—Figure 4), diagnostic models (Figure 5A,B), intraoral scans (Figure 5C,D), and dental photography (Figure 6, Figure 7, Figure 8, Figure 9 and Figure 10) are used.

While traditional radiography remains a cornerstone in the initial assessment, the advent of cone-beam computed tomography has revolutionized the diagnostic process. CBCT offers three-dimensional visualization of the tooth and surrounding structures, providing unparalleled insights into the extent of ankylosis, the condition of the alveolar bone, and the presence of any root abnormalities or pathologies that could complicate treatment. This imaging modality is particularly beneficial in planning surgical interventions and orthodontic management, allowing for a more precise and tailored approach to treatment in complex and severe cases. Furthermore, recent developments in digital dentistry, including intraoral scanning and digital occlusal analysis, have enhanced the ability to monitor the progression of infraocclusion and its effects on the dental occlusion and adjacent teeth. These technologies facilitate a more dynamic and accurate assessment of the tooth’s position relative to the occlusal plane and the surrounding dentition, enabling early intervention and more predictable treatment outcomes. The radiological image shows discontinuity or narrowing of the periodontal space, increased density of the bone adjacent to the affected tooth, asymmetric root resorption in primary teeth, and displacement, delay in growth, or root bending in the permanent ones. Histopathological examination reveals discontinuity of Hertwig’s epithelial root sheath and segmental loss of periodontal fibers. Regular evaluation of the position of adjacent and opposite teeth and the measurements of the distance between the occlusal plane of the reincluded tooth and the occlusal plane of the adjacent teeth are very helpful in monitoring the progress of the tooth submerging, as well as in creating a treatment plan. These measurements can be performed directly in the mouth or indirectly using diagnostic models, intraoral scans (Figure 5), or dental photography (Figure 6, Figure 7, Figure 8, Figure 9 and Figure 10) [4,10,23,24,25,26].

The management of submerged teeth is highly individualized, depending on the extent of infraocclusion, the presence of associated dental or skeletal anomalies, and the patient’s overall oral health status. Treatment objectives focus on preventing or mitigating complications such as malocclusion, adjacent tooth displacement, and functional impairments.

Observation is the most frequently recommended procedure for primary teeth with partial reinclusion (Figure 6A). A significant progression of the submerging process is often observed during the pubertal growth spurt. If ankylosis occurs early, the reinclusion is expected to progress. Concominantly the progression of infraocclusion is expected to be low after cessation of growth [27,28]. If the infraocclusion does not progress, such a tooth should be left for its physiological exfoliation and natural replacement. Concomitantly, deciduous molar infraocclusion is associated with distoangulation of mandibular second premolar germ, which could lead to lack of spontaneous eruption of the permanent successor. Other factors associated with lower second premolar distoangulation are agenesis of its antimere and small maxillary lateral incisors [29]. Any treatment is implemented after thorough diagnostics by a multidisciplinary team (orthodontist, oral surgeon, conservative dentist, prosthodontist, and periodontist) [5].

If the reincluded tooth is not mobile at the time of natural replacement, a permanent successor is present, and a single tooth is present in the oral cavity on the opposite side for at least 6 months, the reincluded tooth should be removed as soon as possible so as not to disturb the eruption of the permanent successor. This protects against incorrect eruption of the permanent successor and reduces the risk of impaction of this tooth. If the tooth is partially reincluded and there are no other disorders, it is possible to level the occlusal plane with the use of composite materials or steel crowns to incorporate the tooth into the occlusion. Moreover, this prevents the Godon effect and the tilting of adjacent teeth towards the reincluded tooth. It should be noted that in patients of developmental age the Godon effect is more complex. It is not just a dental problem involving disruption of the occlusal plane. In patients in the growth phase, the alveolar process also grows, and the disorder also affects the alveolar bones. The alveolar ridge is higher in the arch opposing the position of the reincluded tooth. Unfortunately, sometimes the patients are diagnosed too late, when the tooth has completely disappeared below the gingival margin, causing the displacement of the permanent successor germ. In such a case, extraction of the affected primary tooth should be performed (Figure 8) and a space maintainer should be considered to save space for its permanent successor (ring-loop maintainer, lingual arch, or Nance plate). A space maintainer is indicated if the expected eruption time of the permanent premolar is longer than 6 months and there is adequate space in the dental arch. If there is not enough space, it should be recreated with the orthodontic appliance before making a retainer. It is reasonable to postpone the extraction if it is necessary to use the tooth as an anchorage in orthodontic treatment (Figure 9) [4,22].

Other factors which should be taken into consideration are the intercuspation of the teeth and the degree of dental development. If it is necessary to remove a primary tooth earlier than the natural exfoliation time (over 2 years), the eruption of its permanent successor should be closely monitored. In the place where the reincluded tooth was located, a thick layer of cortical bone may form, which will cause difficulties in erupting the permanent tooth. The tooth may be stopped or have its eruption delayed. In such cases, shortening of the roots of the permanent successor is also observed. If there are no obstacles to the eruption of a permanent successor (such as a thick cortical layer or lack of space in the dental arch), then the vertical bone deficiency resulting from infraocclusion and extraction of a primary tooth is automatically eliminated after the eruption of a premolar [4,22].

Maintaining a deciduous molar in patients with agenesis of the premolar successor is a reasonable treatment option. Clinical monitoring is required to evaluate mobility and the progression of infraocclusion. This should be associated with radiographic examination every 6 months to assess root resorption [29,30]. However, infraocclusion could cause reduced vertical growth in the area. In case of the absence of a permanent tooth germ, the tooth with progressive infraocclusion should be removed before a significant vertical disturbance of the occlusal plane occurs and a defect in the alveolar bone develops (Figure 10) [28,31]. Bone loss resulting from the extraction leads to less serious disorders than the possible defect due to progression of infraocclusion. Some authors suggest considering extraction of deciduous teeth without successors and showing infraocclusion of more than 2–3 mm before the growth spurt [27]. If it is necessary to use the tooth as an anchorage in orthodontic treatment, it is reasonably justified to postpone the extraction if it necessary to use the tooth as an anchorage in orthodontic treatment (Figure 9) [4,22]. During the orthodontic treatment, closing the space or replacing the missing tooth should be considered. The type of procedure depends on the amount of space in the arch, occlusal relationships, the profile, and the patient’s individual growth pattern [4,5,15,32].

For the permanent teeth, there is no single treatment protocol. Initially, in the case of partial infraocclusion, the progress of submerging of the affected tooth should be monitored or orthodontic extrusion should be attempted without or with surgical subluxation of the tooth. If the tooth crown is visible in the oral cavity, no surgery is needed. If the tooth is completely impacted, a mucoperiosteal flap should be made and an orthodontic abutment should be cemented. The tooth should be concominantly subluxated. An additional corticotomy may be performed to facilitate the process of guiding the tooth into occlusion. Corticotomy involves a vertical incision of the cortical bone relative to the surface of the tooth root. The aim of the procedure is to reduce bone resistance when moving teeth during orthodontic treatment [33]. Orthodontic extrusion involves applying force using an elastic traction to achieve tooth movement towards the occlusal plane [4,22]. The use of orthodontic skeletal anchorage (orthodontic microimplants and miniplates) should be considered to reduce undesirable side effects, such as a displacement of teeth that are the traditional source of anchorage [24].

Other surgical methods include decoronation, i.e., cutting off the tooth crown. This is an often-recommended procedure in the treatment of ankylosed teeth with progressive infraposition. The technique is to remove the crown of the tooth and to leave the ankylosed root and the alveolus to be replaced by bone. Only teeth without infection and periapical lesions can be subjected to the decoronation procedure. The crown of the tooth should be cut off to remove all the enamel. The root filling should be removed [34]. The coronal part of the root surface must be reduced to a level of 1.5–2.0 mm below the marginal bone level. Then, the mucoperiosteal flap should be mobilized and the wound closed. It has been proven that within 2–3 years of the procedure, the alveolar ridge grows by 1 mm in the vertical direction [35]. This is probably due to the formation of new bone and periodontal fibers over the remaining root. New periodontal fibers are connected to the adjacent teeth. In patients of developmental age, further tooth eruption occurs next to the decorated tooth. This creates a pulling force on the tooth, which causes further growth of the alveolar ridge. This allows the width and height of the alveolar ridge to be maintained until treatment with dental implants is implemented [36].

In the case of severe infraocclusion, the treatment of choice is tooth extraction. The procedure can be very complicated due to the tilt of the teeth relative to the gap and the difficult access, as well as root dilaceration. The adjacent teeth could be uprighted with an orthodontic appliance to make the surgical procedure easier. Often, the procedure requires coronal–root separation of the tooth, mucoperiosteal flap elevation, and osteotomy [37,38,39,40]. In the case of reincluded teeth into the maxillary sinus, it may open, and cause complications related to the maxillary sinus, such as the oro–sinus communication, oro–sinus fistula, or maxillary sinusitis. The roots of submerged teeth in the mandible may be located deep in the body of the mandible and surrounding or entwining the inferior alveolar nerve canal or the lower border of the mandible. If it is necessary to remove a tooth, it may result in disruption of the neurovascular bundle, bleeding, damage to the inferior alveolar nerve, or sensory disturbances. If there is significant bone loss during the extraction of such a tooth, the area may require miniplate osteosynthesis. This is a place of reduced resistance and may result in a fracture of the mandible [37,38,39,40,41,42,43].

The field of regenerative dentistry holds promise for treating tooth infraocclusion, particularly through the development of biomaterials that can stimulate bone regeneration and tooth movement. Techniques such as low-intensity pulsed ultrasound (LIPUS) have shown potential in promoting bone growth and could be applied to encourage the upward movement of submerged teeth. Similarly, the application of platelet-rich plasma (PRP) and stem-cell therapies are being explored for their regenerative capabilities in oral tissue, offering hope for less invasive and more effective treatment options [44].

In cases where the reincluded tooth requires removal, implant treatment can be performed after bone growth has finished. Concomitantly, in patients of developmental age and in adults, tooth autotransplantation may be an alternative. The procedure involves transplanting another tooth in place of the reincluded tooth. The procedure can be planned based on a CBCT scan and a replica of the transplanted tooth prepared before the procedure. A tooth replica can be made using a 3D printer or milled in a prosthetic laboratory. During the surgical procedure, a new socket must be prepared using a tooth replica. Once the new socket is prepared, the donor can be removed. Donor extraction must be gentle, and the periodontal ligament cannot be damaged. After the procedure, the tooth should be splinted semi-epistically with sutures or using a splint, e.g., a titanium trauma splint (TTS). Fixation should be maintained for 2 weeks. If there is high mobility, immobilization should be maintained. It is assumed that after 4 weeks the soft tissue is completely healed, and new bone can be observed in radiological examination around the tooth 3–4 months after the procedure. Teeth with incomplete root growth have a better prognosis. Therefore, if a tooth that is planned to be transplanted has a completely formed root, the endodontic treatment should be performed [45,46].

## 3. Discussion

Dental infraocclusion, a complex phenomenon affecting both primary and permanent dentition, emerges as a secondary process where teeth initially well-positioned within the dental arch sink below the occlusal plane. This regression, often attributed to a constellation of periodontal and bone pathologies, signifies a post-eruptive downward movement distinct from impaction, where teeth remain unerupted primarily due to spatial constraints. Despite the anatomical differences between primary and permanent teeth, the etiological factors precipitating reinclusion demonstrate remarkable similarity. Understanding these underpinnings is crucial for delineating effective management strategies, emphasizing the need for comprehensive diagnostics to distinguish between infra-occlusion and impaction [1,2]. It is suggested that infraocclusion of primary molars may be considered an early marker for the development of later-appearing dental anomalies. The presence of infraocclusion was associated with tooth agenesis, microdontia of maxillary lateral incisors, palatally displaced canines, and distal angulation of the mandibular second premolars [1].

The findings of this narrative review are consistent with existing literature, which similarly emphasizes the heterogeneity of tooth submerging’s etiology and the critical role of early and accurate diagnosis in managing the condition effectively. However, this article extends the discourse by incorporating recent advancements in diagnostic and treatment technologies, offering fresh insights into potential therapeutic strategies.

The article’s insights into the diagnostic and therapeutic approaches underscore the necessity of a tailored, interdisciplinary management strategy. This approach not only addresses the immediate concerns associated with submerged teeth but also mitigates long-term complications, such as malocclusion and adjacent tooth displacement, thereby preserving oral health and functionality.

Despite the advancements in understanding and managing tooth submerging, several areas require further investigation. Future research should aim to elucidate the genetic and molecular underpinnings of ankylosis and bone inertia, offering potential for preventive strategies. Additionally, longitudinal studies assessing the outcomes of various treatment modalities could provide valuable data to refine therapeutic protocols. The exploration of minimally invasive and regenerative treatment options, including the use of growth factors and stem cell therapy, represents another promising avenue for research [44].

## 4. Conclusions

This article has elucidated the multifaceted nature of infraocclusion, emphasizing its prevalence, diagnostic challenges, and the necessity for timely, individualized treatment approaches. We underscored the importance of an interdisciplinary team in assessing and managing infraocclusion, reflecting on the spectrum of interventions from observation to surgical extraction, based on severity and the specific dental architecture of the patient. Treatment of mild reinclusion often involves observation in both the primary and permanent dentition. Treatment procedures in moderate infraocclusion include observation and reconstruction of the occlusal plane height. If the pathology progresses, which is often observed during the pubertal growth spurt, the tooth should be removed. In the permanent dentition, a moderately reincluded tooth can be pulled using orthodontic extrusion forces. Severe reinclusion requires tooth extraction due to angulation of the adjacent teeth with loss of space, both displacement and growth impairment of the successor teeth, vertical disturbance of the occlusal plane, and increasing bone defects. Deciduous teeth without permanent successors deserve special attention. After extraction, the space can be recreated or maintained with an orthodontic appliance. Then, the missing tooth can be replaced with autotransplantation or an implant. Alternatively, the space could be closed during the orthodontic treatment. This comprehensive analysis aims to advance the understanding of infraocclusion, fostering improved patient outcomes through early intervention and tailored therapeutic strategies.

## Data Availability

The data presented in this study are available on request from the corresponding author. The data are not publicly available due to privacy restrictions.

## Abbreviations

CBCT	Cone beam computed tomography
GAPO syndrome	Growth delay–alopecia–pseudoanodontia–optic atrophy syndrome
LIPUS	Low-intensity pulsed ultrasound
MFE	Mechanical failure of eruption
PFE	Primary failure of eruption
Phase A1	The tooth crown reaches half the height of the crowns of adjacent teeth
Phase A2	The crown of the embedded tooth is located between half the height of the crowns of adjacent teeth and the cervix
phase B	The tooth crown is below the oral mucosa
PRP	Platelet-rich plasma
TTS	Titanium trauma splint
36	Permanent lower first molar
55	Primary upper right second molar
74	Primary lower left first molar
75	Primary lower left second molar
84	Primary lower right first molar
85	Primary lower right second molar
